# The effect of blood storage age on treatment of lactic acidosis by transfusion in children with severe malarial anaemia: a pilot, randomized, controlled trial

**DOI:** 10.1186/1475-2875-12-55

**Published:** 2013-02-06

**Authors:** Aggrey Dhabangi, Edison Mworozi, Irene R Lubega, Christine M Cserti-Gazdewich, Albert Maganda, Walter H Dzik

**Affiliations:** 1 Child Health and Development Centre, Makerere University, Kampala, Uganda; 2 Department of Paediatrics and Child Health, Mulago Hospital, Ministry of Health Kampala, Kampala, Uganda; 3 Department of Hematology, University of Toronto, Toronto, Canada; 4 Baylor College of Medicine Children’s Foundation, Kampala, Uganda; 5 Blood Transfusion Service, Massachusetts General Hospital, Boston, USA

**Keywords:** Severe malarial anaemia, Lactic acidosis, Blood storage age, Blood transfusion, Children

## Abstract

**Background:**

Severe malarial anaemia requiring blood transfusion is a life-threatening condition affecting millions of children in sub-Saharan Africa. Up to 40% of children with severe malarial anaemia have associated lactic acidosis. Lactic acidosis in these children is strongly associated with fatal outcomes and is corrected by blood transfusion. However, it is not known whether the storage age of blood for transfusion affects resolution of lactic acidosis. The objective of this pilot study was to evaluate the effect of blood storage age on resolution of lactic acidosis in children with severe malarial anaemia and demonstrate feasibility of conducting a large trial.

**Methods:**

Children aged six to 59 months admitted to Acute Care Unit of Mulago Hospital (Kampala, Uganda) with severe malarial anaemia (haemoglobin ≤ 5 g/dL) and lactic acidosis (blood lactate ≥5 mmol/L), were randomly assigned to receive either blood of short storage age (one to 10 days) or long storage age (21–35 days) by gravity infusion. Seventy-four patients were enrolled and randomized to two equal-sized study arms. Physiological measurements, including blood lactate, oxygen saturation, haemoglobin, and vital signs, were taken at baseline, during and after transfusion. The primary outcome variable was the proportion of children whose lactic acidosis resolved by four hours after transfusion.

**Results:**

Thirty-four of 37 (92%) of the children in the short storage treatment arm compared to 30/37 (81%) in the long storage arm achieved a blood lactate <5 mmol/L by four hours post transfusion (p value = 0.308). The mean time to lactic acidosis resolution was 2.65 hours (95% CI; 2.25–3.05) in the short storage arm, compared to 3.35 hours (95% CI; 2.60–4.10) in the long storage arm (p value = 0.264).

**Conclusion:**

Pilot data suggest that among children with severe malarial anaemia and lactic acidosis transfused with packed red blood cells, the storage age of blood does not affect resolution of lactic acidosis. The results support a larger and well-powered study which is under way.

**Trial registration:**

clinicaltrials.gov NCT01580111

## Background

Severe malarial anaemia requiring blood transfusion remains a major health issue in sub-Saharan Africa [1]. It is the commonest defining manifestation of severe malaria among children at Acute Care Unit (ACU) of Mulago Hospital (Kampala, Uganda), occurring in 39.4% of children with malaria [2]. Severe malarial anaemia causes a substantial reduction in the oxygen-carrying capacity of blood, such that despite increases in cardiac output, some affected children develop inadequate tissue oxygenation and tissue lactic acidosis (LA) [3].

Lactic acidosis, defined by the World Health Organization as a blood lactate level >5 mmol/L, is found in approximately 41% of children with severe malarial anaemia [3]. Lactic acidosis is directly associated with fatal outcomes in malaria and is present in 75% of fatal cases [3,4]. Indeed, LA is more pronounced in severe malarial anaemia compared with severe non-malarial anaemia and case fatality rates may be up to twice as high (9% *vs* 4%) in malaria for the same level of anaemia [5].

Previous studies in a limited number of patients have documented that blood transfusion resolves the LA associated with severe malaria anaemia. English *et al.* reported correction of high lactate levels (mean = 11.2 mmol/L) in 16 children with *Plasmodium falciparum* malaria who had haemoglobin levels <5 g/dL. Whole blood transfusion resulted in correction of elevated lactates in 80% of children within four hours [6]. The same authors subsequently reported results on nine additional children whose lactic acid levels fell with transfusion by 45% from a mean (± SD) of 8 ± 4.7 mmol/L (pre-transfusion) to 3.6 ± 3.5 mmol/L following whole blood transfusion [7]. In a preliminary study of 18 children with severe malaria anaemia, it was reported that transfusion resulted in a 45% increase in Hb concentration and a 46% decrease in blood lactate levels within three to five hours after transfusion [8]. No previous studies have examined the effect of blood storage on resolution of LA in malaria.

Nearly all transfusions are administered after a period of refrigerated blood storage. During storage, red blood cells are known to undergo biochemical and structural changes, which have been postulated to impair oxygen delivery to tissues [9]. There are no prior studies on the clinical effects of blood storage in patients with severe malaria anaemia and no prospective studies on blood storage in subjects with profound anaemia and insufficient oxygen-carrying capacity as evidenced by LA [9-12]. Given the fatality of untreated severe malaria anaemia accompanied by LA and given the practical requirements for blood storage prior to transfusion, the question of whether the storage age of blood affects oxygen delivery to tissues and/or clinical outcomes is of vital importance in the treatment of children with severe malaria anaemia. Given the concerns raised about the potential toxicity of stored blood and the practical difficulties of conducting a randomized clinical trial of blood storage age in acutely ill children, this current pilot study was performed. It was designed to compare the two extremes of blood storage in children with severe malaria anaemia and LA. The study was based on the hypothesis that there is no difference between those transfused with blood of either long or short storage age.

## Methods

The study was a pilot, prospective, randomized, open-label clinical trial, conducted between December 2010 and August 2011 at the Acute Care Unit (ACU), the paediatric emergency unit of Mulago Hospital, the national referral hospital in Kampala, Uganda. Children aged six months to 59 months were enrolled if they fulfilled all the following criteria: i) a positive blood smear for malaria, ii) severe anaemia (Hb ≤5 g/dL), iii) lactic acidosis (blood lactate ≥5 mmol/L), and iv) written informed consent from the parent or guardian. Children with known or concurrent cardiac disease and those undergoing transfusion with blood products other than packed red cells were excluded.

Verbal screening consent to obtain a blood lactate was initially sought from the caretaker in the emergency room to identify those with lactate ≥5 mmol/L who fulfilled the inclusion criteria. Full written consent from the caretaker was done later after resuscitating the child. Blood smear for malaria and haemoglobin estimation are routine tests at the paediatric emergency unit of Mulago Hospital. Eligible children were randomized using the sealed envelopes method into two treatment arms: short storage arm (one to 10 days) and long storage arm (21–35 days).

All children were transfused with packed red blood cells (RBCs) supplied directly from the Uganda Blood Transfusion Service centre based at Nakasero, Kampala. The transfusion centre collects volunteer donor blood and routinely tests all donated blood for HBsAg and for antibodies to HIV, HCV and syphilis within twenty four hours prior to release to the hospital. Blood was stored as packed RBCs in SAGM anticoagulant-preservative solution with an expiration date of 35 days from the date of collection. The blood was kept at the ACU blood bank, refrigerated at temperature ranges of 4-8°C. When multiple blood units were available for a particular treatment arm, the actual unit to be transfused was selected at random. Blood transfusions were according to local standard practice which is (approximate) 10 ml of packed RBCs per kg body weight of the child; transfused over 50–60 min by gravity infusion. Children were transfused with compatible blood after grouping (ABO and Rhesus groups) and cross-matching. The volume of blood transfused was computed using its weight (bag weight at pretransfusion minus its post transfusion weight, divided by the specific gravity of blood (1.06)).

The study variables included: sociodemographic characteristics, laboratory (blood smear for malaria parasites and quantitative parasite count), and clinical data: blood pressure (Omron 7051T), heart rate, oxygen saturation (Nonin Medical Inc, USA, Model 2500), respiratory rate, capillary blood lactate (Lactate Pro, Arkray, Japan), and haemoglobin (Hemocue, Angelholm, Sweden). Values were recorded on a standardized case report form at baseline and follow up at 2, 4, 6, 8, 12, and 24 hours post-transfusion until lactate levels fell to <5 mmol/L. The outcome measure was the proportion of transfused children who resolved lactic acidosis by four hours from the start of the transfusion. Apart from blood transfusion, all enrolled children received routine standard treatment for severe malaria with intravenous quinine. Apart from intravenous quinine given in 5% dextrose, children received no additional fluid therapy.

Data was double entered using EPI –DATA (version 3.2), exported to SPSS (version 12) for analysis. Analysis was done by intention to treat. The proportion of children from each group who achieved the primary outcome was compared using the Chi-squire test for categorical variables. The student’s t-test was used for comparing means of continuous variables of normal distribution. The Kaplan Meier survival curve was used to analyse differences in time to LA resolution between the two groups. Significance was assigned to p-values <0.05.

Quality control measures included: the case report form for data collection was pre-tested by the principal investigator on five subjects; the research assistants were trained on data collection procedures; the blood lactate meter was calibrated at least twice a month using the disposable calibration strip that came from inside of each packet of twenty five strips; and, measurements of blood pressure, respiratory rate and weight of child were taken twice, and the average of the two readings recorded.

Parents or guardians of participating children provided written informed consent and were free to voluntarily withdraw at any time. Confidentiality and participant anonymity were explained to those providing consent. Permission to carry out the study was obtained from the Department of Paediatrics and Child Health of the College of Health Sciences. The study was approved and cleared by the institutional Review Committee (IRC) of the College of Health Sciences, Makerere University. The trial was registered (NCT01580111).

A Data and Safety Monitoring Board (DSMB) composed of three senior faculty members (a paediatrician, a statistician and a haematologist) oversaw ethical conduct of this trial. Any serious adverse event was recorded and reported to the DSMB within 24 hours of occurrence. The DSMB reviewed study progress after 25% and 50% recruitment and at the end of the study. No difference in the rate of adverse events in the two treatment arms was observed.

## Results

### Patient recruitment and baseline characteristics

**Figure 1:**
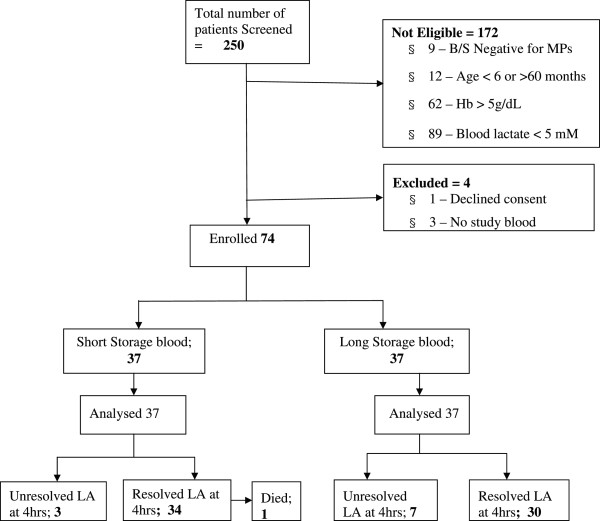
Profile of study participants with severe malarial anaemia and lactic acidosis at Mulago Hospital during Dec 2010 – Aug 2011.

A total of 250 children were screened for enrolment. Lactate levels >5 mmol/L were observed in 78 of 167 (46.7%) of children with severe malaria anaemia (haemoglobin levels <5 g/dL). Of these, 74 children requiring blood transfusion were enrolled and randomly assigned to receive either blood of short storage or long storage (see Figure 1). Apart from sex, most baseline characteristics were distributed evenly in both study groups as shown in Table 1.

**Table 1:** Baseline characteristics of study participants with severe malarial anaemia and lactic acidosis at Mulago hospital; n = 74

	**Treatment arm; n (%)**			
**Variable**	**Short storage**	**Long storage**	**Total**	**P-value**
Sex = Female, n (%)	22 (59.5%)	14 (37.8%)	N/A	0.063
Age (months), mean (SD)	27.6 (16.6)	23.1(15.2)	N/A	0.225
Weight (Kg), mean (SD)	10.3 (3.3)	10.3 (2.8)	N/A	0.931
Height (cm), mean (SD)	82.4 (13.3)	81.6 (12.7)	N/A	0.798
**Caretaker** Mother, n (%)	28 (75.7%)	25 (67.6%)	**53**	0.439
Father, n (%)	2 (5.4%)	9 (24.3%)	11	0.022
Others, n (%)	7 (18.9%)	3 (8.1%)	10	0.174
**Co-infections,** n (%)	7 (18.9%)	7 (18.9%)	14	1.000
**Parasitaemia score at ACU**	**37 (100%)**	**37 (100%)**	74	
1+	7 (18.9%)	6 (16.2%)	13 (17.6%)	0.760
2+	11 (29.7%)	18 (48.6%)	29 (39.2%)	0.096
3+	12 (32.4%)	8 (21.6%)	20 (27%)	0.295
4+	7 (18.9%)	5 (13.5%)	12 (16.2%)	0.528
**ABO blood group of child**	**37(100%)**	**37(100%)**	**74**	
O	19(51.4%)	13(35.1%)	32(43.2%)	0.159
A	7(18.9%)	13(35.1%)	20(27%)	0.116
B	8(21.6%)	9(24.3%)	17(23%)	0.782
AB	3(8.1%)	2(5.4%)	5(6.8%)	0.643
**Quantitative parasite count**	Median (IQR)			
Median parasite count /μL	19,760 (9,680 – 78,120)*	18,440 (3,200 – 57,600)*		0.106

*Data is not normally distributed.

Most baseline clinical parameters show that the study children were very sick. The mean pretransfusion haemoglobin was 4.0 (±0.9) g/dL and 3.7 (±0.8) g/dL, while the mean blood lactate was 9.2 (±2.8) mmol/L and 9.4 (±2.9) mmol/L in the short storage and long storage arm respectively. These parameters were comparable in both study arms. The means of the other pretransfusion physiological parameters, like oxygen saturation, respiratory rate, heart rate, blood pressure are shown in Table 2.

**Table 2:** Baseline clinical characteristics of children with severe malarial anaemia and lactic acidosis at Mulago hospital; N = 74

	**Treatment arm of the study**		
**Variable**	**Mean (SD)**		**P-value**
	**Short storage (n = 37)**	**Long storage (n = 37)**	
Haemoglobin (g/dL)	4.0 (0.9)	3.7 (0.8)	0.134
Blood lactate (mmol/L)	9.2 (2.8)	9.4 (2.9)	0.764
SPO2 (%)	96.8 (3.1)	96.5 (4.5)	0.739
Respiratory rate(/min)	60 (18.0)	57 (14.6)	0.434
Heart rate (beats/min)	156 (17.2)	161 (14.0)	0.175
Systolic BP(mmhg)	91.2 (14.2)	91.2 (16.5)	1.0
Diastolic BP (mmhg)	52.9 (11.8)	52.0 (14.3)	0.769
WBC x10^3^(/μl)	14.9 (16)	14.8 (8.7)	0.973
Neutrophil count x10^3^(/μl)	7.3 (7)	5.2 (2.7)	0.093
Serum Na + (mmol/L)	137.4(5.2)	139.7(7.2)	0.119
	**Median (IQR)**		
Platelet count x10^3^(/μl)	91 (64–183) *	103 (69 – 166.5)*	0.528

*Data is not normally distributed.

The mean storage age of blood in the short storage arm was 7.8 (±1.8) days, compared with 27.2 (±3.9) days in the long storage arm. The volumes of blood transfused were similar in both study groups, namely; 12.7 (±2.6) ml/kg and 12.7 (±2.2) ml/kg in the short and long storage arms respectively. Similarly, the duration of blood transfusion had a median (IQR) of 51 (45–55) min and 53 (47–57) min for the short and long storage age respectively (see Table 3).

**Table 3:** Interventional characteristics of children with severe malarial anaemia and lactic acidosis at Mulago hospital; N = 74

**Variable**	**Short storage arm** **Mean (SD)**	**Long storage arm** **Mean (SD)**	**P-value)**
Volume of blood transfused (ml)	125.4 (±28.9)	127.1 (±27.4)	0.796
Volume of blood transfused ml/kg	12.7 (±2.6)	12.7 (± 2.2)	0.321
Duration of blood transfusion (min)	50 (±5.9)	51 (±7.1)	0.512

### Proportion of patients who resolved lactic acidosis by four hours after transfusion

Of the 74 children with severe malarial anaemia and LA who were enrolled and transfused, 64 of 74 achieved the primary outcome of a blood lactate <5 mmol/L within four hours of transfusion. Resolution of LA within four hours occurred in 34 of 37 (92%) in the short storage study group compared to 30 of 37 (81%) in the long storage group. This difference was not statistically significant (p value = 0.308; Fisher’s exact test).

### Time to resolution of lactic acidosis

**Figure 2:**
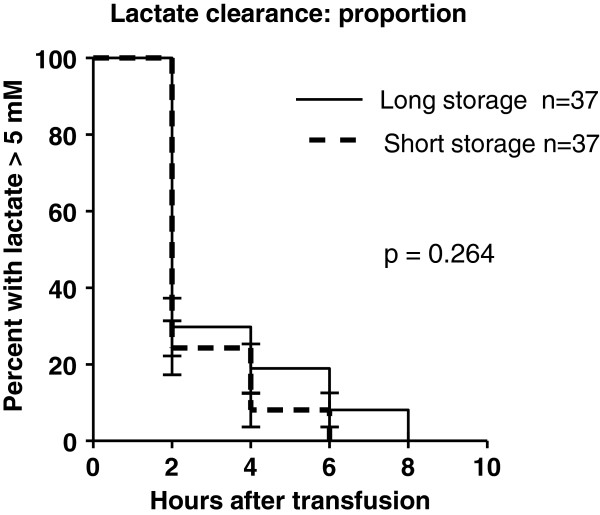
Kaplan- Meier curve showing time to lactic acidosis resolution among children with severe malarial anaemia and lactic acidosis; N=74.

The time taken to achieve the primary outcome (time to event) was determined using Kaplan-Meier analysis. In all 74 patients transfused, LA had resolved at the eight-hour mark at follow up, regardless of the treatment arm. The mean time to LA resolution was 2.65 hrs (95% CI; 2.25–3.05) in the short storage arm, compared to 3.35 hrs (95% CI; 2.60–4.10) in the long storage arm (see Figure 2). The log rank analysis showed no statistical significance (p-value = 0.264).

### Changes in the vital signs following transfusion

**Figure 3:**
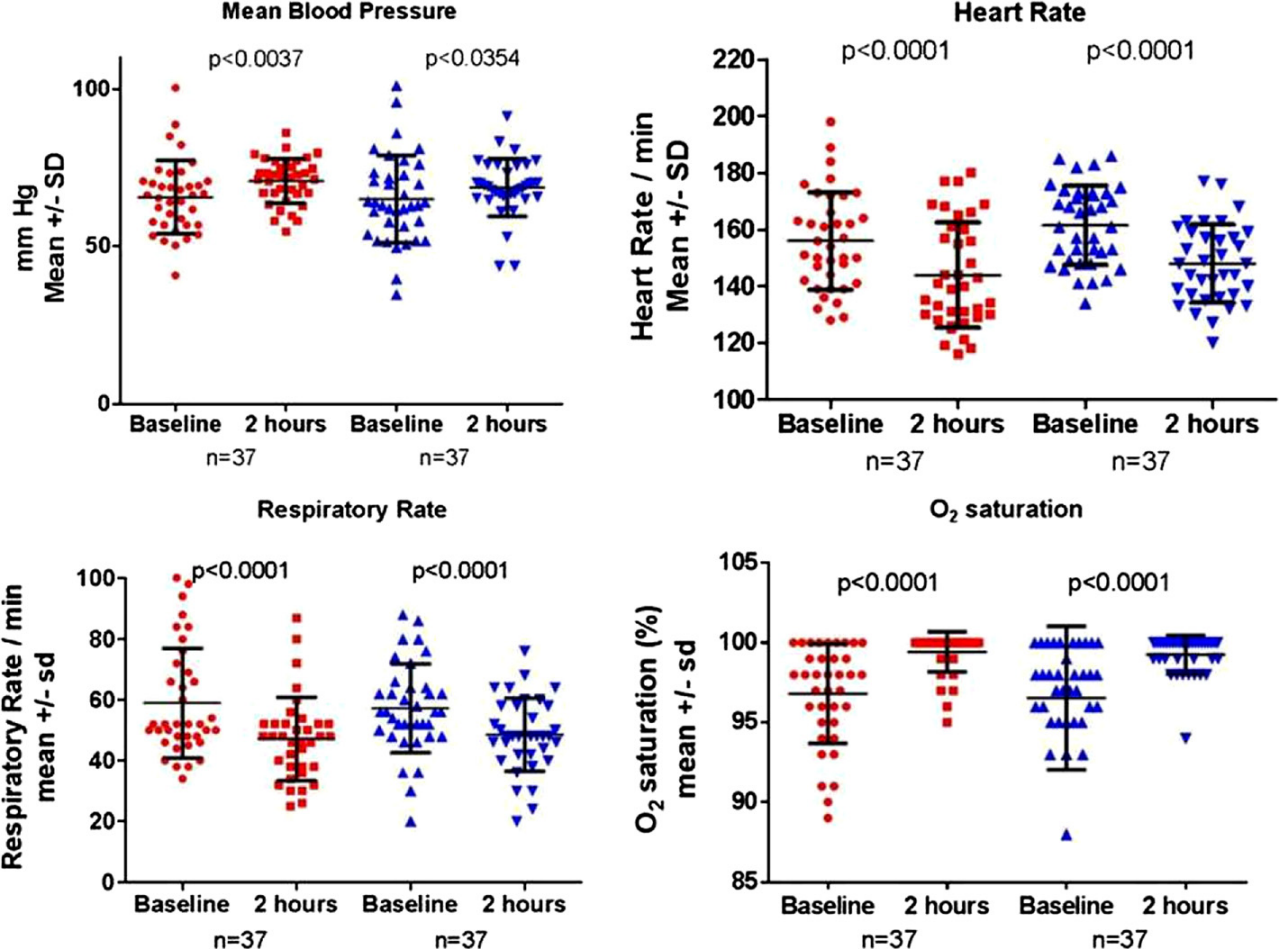
**Vital signs before and after transfusion among children severe and lactic acidosis at Mulago Hospital during Dec 2010 – Aug 2011.** Each panel shows the mean and SD for vital sign values before transfusion and two hours after the start of the transfusion. Data in red indicates the short-storage arm and blue indicates the long storage arm.

Following blood transfusion, there were remarkable improvements in the baseline clinical parameters in all children. The respiratory rate and the heart rate improved at two hours compared to baseline and the oxygen saturation, mean blood pressure too improved (see Figure 3).

### Adverse events

During the 24 hours of follow up, three adverse events were reported; with two out of three occurring in the short storage arm compared to one out of three in the long storage study arm. Two children (one in either study arm) had seizures within four hours after transfusion. The third event was a death that occurred three hours after transfusion.

## Discussion

This pilot clinical trial was conducted to examine the feasibility of performing a larger study on the clinical effect of blood storage in profound malarial anaemia. The goal of this pilot study was to compare proportions of children aged six to 59 months with severe malarial anaemia that resolved LA by fours following the start of transfusion with packed RBCs of long storage (21–35 days) versus short storage age (one to 10 days). The study found that 92% of the children transfused with blood of short storage compared to 81% with long storage had resolved their LA at four hours post transfusion, a difference which was not statistically significant. Similarly, there was no significant difference between short storage and long storage blood for the time taken to resolve LA. These results suggest that in the treatment of severe malarial anaemia (SMA) complicated with LA, the storage age of blood for transfusion does not affect LA resolution.

This study is the largest study on the clearance of hyperlactataemia by transfusion in children with malaria and the first to evaluate the influence of blood storage age on resolution of malarial LA. Indeed research on resolution of LA by transfusion is scarce despite severe anaemia being a leading cause of death in children with malaria [3,4]. Two related observational studies published more than a decade ago by English *et al.* at Kilifi, Kenya, demonstrated that transfusion was associated with faster amelioration of respiratory distress and resolution of LA among children with severe malaria anaemia [6,7]. These studies however did not formally evaluate the effect of duration of transfusion, rate of transfusion, or storage age of blood on the resolution of LA. The two studies measured venous blood lactate levels post transfusion in nine and 16 patients respectively. The children were transfused with whole blood rather than packed RBCs and in the second study [6], children also received additional fluids (normal saline) during their resuscitation. In the current study, 16% of children received an additional transfusion within 24 hours of admission, two thirds of whom had not achieved a haemoglobin above 5 g/dL following the first unit transfused. Finally, the proportion of children who resolved an elevated lactic acid level at four hours after transfusion was significantly lower in the study by English (nine of 16) compared with the current study (64 of 74), p <0.0105. In this study, 100% of the 74 transfused children had resolved LA eight hours post transfusion compared with English’s study where two of the 16 children still had unresolved LA at the eight-hour mark despite blood transfusion and additional intravenous normal saline.

Although no single trial has compared blood transfusion *versus* blood transfusion plus fluids in the treatment of SMA and LA, the findings of English *et al.*[6], together with the results of the FEAST trial by Maitland *et al.*[13] suggest that additional intravenous fluids provide no added benefit in the treatment of SMA complicated by LA. In addition, since previous studies on the resolution of LA following transfusion have used either whole blood or packed red cells, the issue of which product is the preferred treatment in children with severe malaria anaemia remains uncertain and requires further research. LA among children with malaria can have multiple causes. While the present study could not prove that the resolution of LA was due to red cell transfusion, the prompt resolution of LA following transfusion suggests that improved tissue oxygen delivery was directly associated with resolution of LA.

Overall, three adverse events were observed within 24 hours of transfusion. One child transfused with short storage RBCs died. Two children, one in each study arm, had generalized tonic-clonic seizures occurring within four hours of transfusion and were managed with rectal diazepam. The fatal case was a young child (seven months old), who besides having SMA and LA also had cerebral malaria and died three hours after blood transfusion, despite having resolved LA at the two-hour mark. Similar findings have been reported by Ranque *et al.*[14] and Cserti-Gazdewich *et al.*[3] who found an increased risk of death among children with multiple co-existing severe malaria syndromes. English *et al.*[6] reported two deaths of the 16 children with SMA and LA, both of whom had hypoglycaemia at admission and one of whom had cerebral malaria (frequent seizures and coma). The present data underscore that some children with malarial anaemia present with severe acidaemia and are in urgent need of red cell transfusion. For such children, adequate blood availability can be life-saving.

This study had several strengths including: screening a large number of potential participants, strict inclusion criteria, uniform assessment, and prompt follow up of the children with LA until resolution. As a pilot trial, this study was not designed to have sufficient statistical power to determine conclusively whether or not blood storage age has an effect on resolution of LA in severe anaemia.

## Conclusion

This pilot trial demonstrated the feasibility and equipoise for a larger definitive study to address the effect of blood storage on the resolution of LA in children with severe malaria anaemia. The results suggest that among children with severe malarial anaemia and LA who are transfused with packed RBCs, the storage age of blood does not affect resolution of LA. A larger and well-powered study on this subject is now underway (NCT01586923). The results of that study should lead to definitive treatment recommendations suitable for sub-Saharan Africa regarding transfusion support for children with severe malaria anaemia and lactic acidosis.

## Abbreviations

SMA: Severe malarial anaemia; LA: Lactic acidosis; RBCs: Red blood cells; ACU: Acute care unit.

## Competing interests

The authors declare that they have no competing interests.

## Authors’ contributions

AD contributed to study design, provided clinical care of the research subjects and follow up, collected clinical data, analysed and interpreted data, and drafted the manuscript. WHD conceived the study, contributed to the study design, helped in the analysis and interpretation of data, and helped draft the manuscript. IRL and EM contributed to study design, provided overall supervision of patient enrolment and helped draft the manuscript. CMC-G participated in study design and helped draft the manuscript. AM participated in the study design, performed the statistical analysis and helped draft the manuscript. All authors read and approved the final manuscript.
